# The Impact of Fluid Loss and Carbohydrate Consumption during Exercise, on Young Cyclists’ Fatigue Perception in Relation to Training Load Level

**DOI:** 10.3390/ijerph18063282

**Published:** 2021-03-22

**Authors:** Leonardo Cesanelli, Berta Ylaitė, Giuseppe Messina, Daniele Zangla, Stefania Cataldi, Antonio Palma, Angelo Iovane

**Affiliations:** 1 Sport and Exercise Sciences Research Unit, Department of Psychological, Pedagogical and Educational Sciences, University of Palermo, I-90128 Palermo, Italy; cesanelli.leonardo@gmail.com (L.C.); daniele.zangla@unipa.it (D.Z.); antonio.palma@unipa.it (A.P.); angelo.iovane@unipa.it (A.I.); 2 Institute of Sport Science and Innovations, Faculty of Sport Biomedicine, Lithuanian Sports University, 44221 Kaunas, Lithuania; berta.ylaite@gmail.com; 3 Department of Basic Medical Sciences, Neuroscience and Sense Organs, School of Medicine, University of Study of Bari, 70122 Bari, Italy; Stefania.cataldi@uniba.it

**Keywords:** fatigue, young athletes, cycling performance, sport nutrition, hydration

## Abstract

High-level young athletes need to face a wide spectrum of stressors on their journey to élite categories. The aims of the present study are (i) to evaluate session rate of perceived exertion (sRPE) at different training impulse (TRIMP) categories and the correlations between these two variables and, (ii) evaluate the correlations between sRPE, fluid loss, and carbohydrate consumption during exercise. Data on Edward’s TRIMP, sRPE, body mass loss pre- and post- exercise (∆), and carbohydrate consumption (CHO/h) during exercise have been acquired from eight male junior cyclists during a competitive season. One-way ANOVA and correlation analysis with linear regression have been performed on acquired data. sRPE resulted in a significant difference in the three TRIMP categories (*p* < 0.001). sRPE resulted in being very largely positively associated with TRIMP values (*p* < 0.001; R = 0.71). ∆ as well as CHO/h was largely negatively related with sRPE in all TRIMP categories (*p* < 0.001). The results confirmed the role of fluid balance and carbohydrate consumption on the perception of fatigue and fatigue accumulation dynamics independently from the training load. Young athletes’ training load monitoring and nutritional-hydration support represent important aspects in athlete’s exercise-induced fatigue management.

## 1. Introduction

High-level young athletes need to face a wide spectrum of stressors on their journey to élite categories [1,2]. Daily training and competitions represent the main physiological load expositions. However, school tasks, social interactions, and the additional physical activities to the training routine, determine a cumulative high-level physio-psychological workload and potentially, an increased fatigue perception [3]. 

Since the balance between stress and recovery has been widely recognized as a key aspect to ensure athlete health and to improve performance, different approaches have been investigated in order to analyze and modulate stressors and recovery factors [2,4]. Monitoring of training stress response represents a fundamental aspect to prevent disruption of this homeostatic balance. The importance of training load monitoring tools has been recently underlined by the observations of Hamlin and colleagues (2019), indicating how physio-psychological load markers may predict young athletes’ injuries as well as a functional tool to manage athlete’s overall stress [2]. Filipas et al., (2019) further reported how acute central fatigue may have a negative impact on endurance performance [5]. This seems to be explained by an increased perception of effort for internal and external loads and, suggesting the importance of fatigue perception monitoring [6,7]. Thus, monitoring of training load and fatigue perception seems to represent valid tools to preserve young athletes’ health and to track and better manage performance fluctuations.

**Figure 1:**
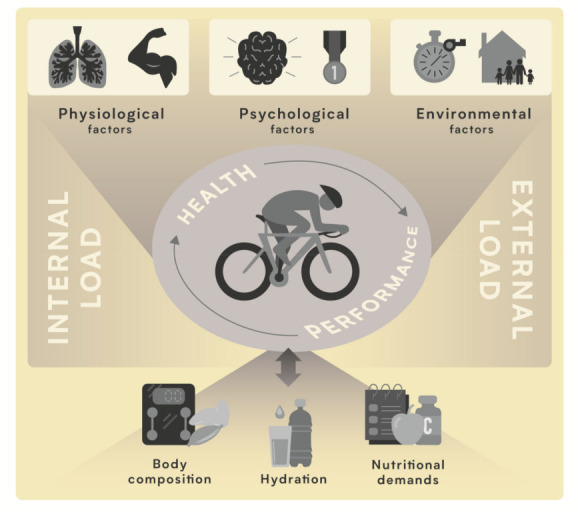
Graphical summary of the main performance and health determining factors of young competitive cyclists.

Nutritional support during exercise represents an additional fundamental aspect to preserve health and performance, with the main aims of covering energetic and plastic demands of physical activity and ensuring a successful recovery [8]. Carbohydrate ingestion during exercise has been associated with improved performance, preventing exercise-induced hypoglycemia, and maintaining high levels of carbohydrate oxidation [8,9,10]. Water has been also suggested as one of the most important ergogenic aids for athletes, with exercising performance described as being significantly impaired when 2% or more body weight is lost through sweat during exercise [9,10]. Further weight loss of more than 4% of body weight has been associated with heat illness, heat exhaustion, heat stroke and possibly death [9,10]. The increased and detailed requirements of young athletes, dictated by the growth process and by the previously described heterogeneity of daily tasks, made nutritional support a focal point in order to ameliorate athlete’s performance and well-being [8]. Fluid intake and hydration status monitoring are also essential factors in young athletes’ growth processes and performance development, as well as in preventing fatigue accumulation [8,11]. Taken together, evidence suggests how young athletes’ training load monitoring and nutritional support are two main aspects to maintain the homeostatic balance between stress and recovery factors (Figure 1).

Thus, the aims of the present study are (i) to evaluate the fatigue perception (session-RPE scale) at different training impulse (TRIMP) categories (low, medium, and high load) and the correlations between these two variables and, (ii) evaluate the correlations fatigue perception (sRPE), fluid loss and carbohydrate consumption during exercise at different training impulse (TRIMP) categories (low, medium, and high load).

## 2. Materials and Methods

### 2.1. Study Participants

Data from a team of 8 male competitive junior category cyclists (16.2 ± 0.7 years; 66.1 ± 4.5 kg; 174.6 ± 4.9 cm) have been acquired during the 2017–2018 season as part of a team health and performance monitoring program. All cyclists performed similar team-monitored training sessions for 2 to 5 days/week (depending on the period of the season and individual training periodization, planned, and prescribed by the team’s staff). All the participants obtained health medical certificates for sport and physical activities as a mandatory procedure to participate in the competitive season. During the investigation period, dietary behaviors, body composition analysis, and training data have been acquired and analyzed by certified sports nutritionists and strength and conditioning coaches. All the participants and families of each participant were fully informed of all aspects of the study and signed a statement of informed consent. This research was designed in accordance with the Declaration of Helsinki (2008), with the Fortaleza update [12].

### 2.2. Measurements

Athletes’ training data were acquired through their personal GPS and HR monitoring cyclocomputers and, successively, exported and analyzed. Based on pre-season testing parameters (i.e., incremental tests and rest HR data) and training data (i.e., HR and duration of training) the Edward’s training impulse (TRIMP) has been calculated for each training as a non-invasive measurement of training load. TRIMP points have been thus obtained as the product of the accumulated training duration (minutes) of five different HR zones, by a coefficient related to each zone (50 to 59% of HRmax × 1; 60 to 69% of HRmax × 2; 70 to 79% of HRmax × 3; 80 to 89% of HRmax × 4, and 90–100% of HRmax × 5), and then summated to obtain the final training load value (i.e., duration in zone 1 × 1 + duration in zone 2 × 2 + duration in zone 3 × 3 + duration in zone 4 × 4 + duration in zone 5 × 5) [13]. The athletes further completed 30 min after each training, the session-RPE scale (BORG-CR10) with values ranging from 0 (no exertion at all) to 10 (maximal exertion) [14]. Body mass was measured using a mechanical balance scale (Seca 874) with a precision of 0.01 kg. Height was measured shoeless using a stadiometer (Seca 213) with a precision of 0.1 cm. The measurements were taken to check the correct position of the head in the standard position of the reference Frankfurt plane [15]. Body mass and height were measured at baseline. In addition, before and after each training session athletes were asked to measure their body mass through the same scale in order to measure the difference in body weight between pre- and post-training (∆) as a non-invasive and easy-to-use fluid loss marker, allowing daily monitoring practices [16]. After each training, athletes reported through a food diary their liquid and/or solid food consumption during the activity [17]. Carbohydrate consumption during exercise (gCHO/h) was then quantified using the Winfood^®^ analysis software. Data have been then categorized according to three training load levels according to TRIMP values: low (<100); medium (100–200); and high (>200). The training sessions has been divided into such categories considering the average characteristics of the training programs prescribed by the team’s staff, respectively, as: recovery training (e.g., 60 min spent in 50–65% HRmax zone), specific training (e.g., 120 min with intervals of high and low intensity according to the target or long-distance low intensity trainings) and high intensity (e.g., competition simulations or real competitions).

### 2.3. Statistical Analysis

All data analyses were carried out using SPSS version 21.0 (IBM Corporation, Armond, NY, USA) and GraphPad Prism version 7.0 (GraphPad Software, San Diego, CA, USA). Descriptive statistics (mean ± SD) were calculated for each variable. Shapiro–Wilk test was used to assess the normality of the samples, revealing normally distributed values. Additionally, the Levene’s test was adopted to assess the homogeneity of the variance for the studied variables indicating a *p* > 0.05. Therefore, One-Way ANOVA was performed to assess the difference in sRPE across the three different training load (TRIMP) categories (low, medium, high). In case of a statistically significant difference, a Tukey post-hoc analysis was applied. Mean difference across pairwise comparison with 95% confidence intervals (95% CI) were also calculated. Additionally, partial eta squared (np2) was used as One-Way ANOVA effect size and interpreted (<0.039—no effect; 0.040 to 0.249—minimum; 0.250 to 0.639—moderate; >0.640—strong [18]). Cohen’s d effect size was established according to the following criteria: 0 to 0.19, trivial; 0.20 to 0.59, small; 0.60 to 1.19, moderate; 1.20 to 1.99, large; 2.00 to 3.99, very large; >4.0; nearly perfect [19]. Pearson correlation analysis and linear regression have been conducted on the data. The following criteria were adopted to interpret the magnitude of correlations between measurement variables: <0.09, trivial; 0.10 to 0.29, small; 0.30 to 0.49, moderate; 0.50 to 0.69, large; 0.70 to 0.89 very large; and >0.90, nearly perfect [19]. An alpha level of *p* ≤ 0.05 was set to assess the statistical significance.

## 3. Results

### 3.1. Fatigue Perception in Different TRIMP Categories and Relationship between TRIMP and sRPE

One-way ANOVA revealed a statistically significant difference (*p* < 0.001; np^2^ = 0.721—strong) between the three investigated training session groups (i.e., <100; 100–200; >200 TRIMP points). Post-hoc analysis indicated significantly lower sRPE (arbitrary unit, AU) in <100 TRIMP training sessions compared with 100–200 (*p* < 0.001; mean difference −1.34 AU) and >200 (*p* < 0.001; mean difference −2.58 AU) and significantly lower sRPE in 100–200 compared to >200 (*p* < 0.001; mean difference −1.24 AU) (Table 1).

**Figure 2:**
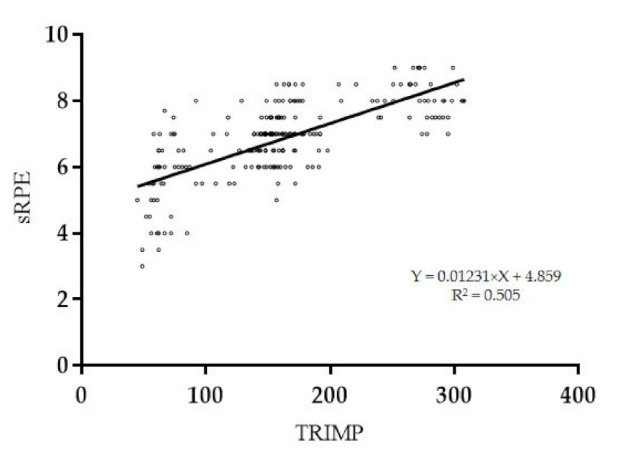
Linear regression between TRIMP (AU) and sRPE (AU).

The Pearson’s r analysis with linear regression between TRIMP (AU) and sRPE (AU) showed a very large positive statistically significant correlation (*p* < 0.001; R = 0.71 (95% CI: 0.641; 0.768); R2 = 0.505) (Figure 2).

### 3.2. Relationships between sRPE and Fluid Loss during Exercise

**Figure 3:**
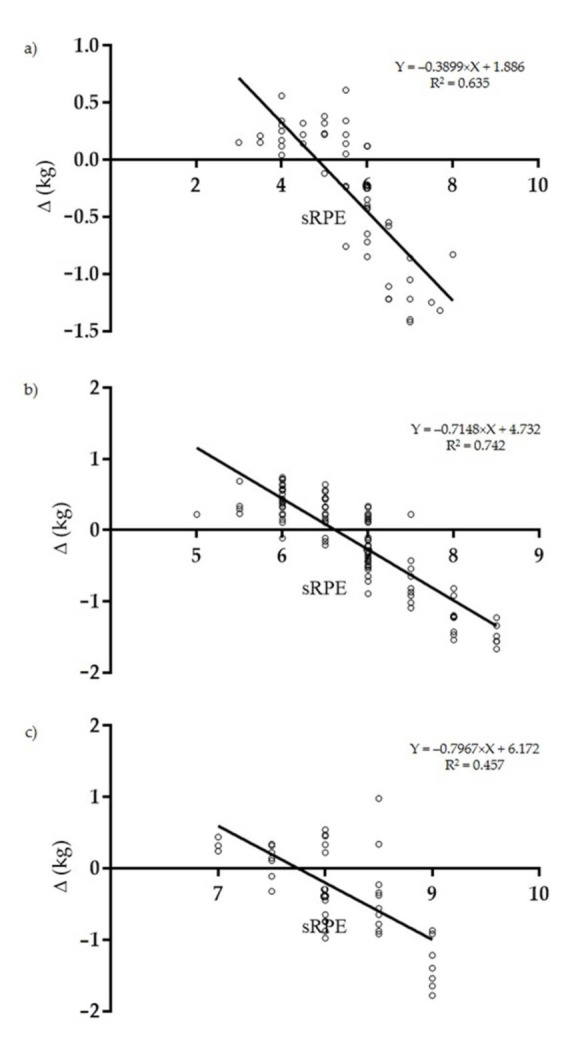
Linear regression between sRPE (AU) and pre- and post-training ∆ (kg) variations of training sessions displaying <100 TRIMP points (**a**); 100–200 TRIMP points (**b**) and >200 TRIMP points (**c**).

Correlation analysis with linear regression of sRPE (AU) and pre- to post-training ∆ (kg) variations revealed a very large negative statistically significant correlation for training sessions with <100 TRIMP points (*p* < 0.001; R = −0.79 (95% CI: −0.87; −0.67); R2 = 0.635) (Figure 3a); a very large negative statistically significant correlation for training sessions with 100–200 TRIMP points (*p* < 0.001; R = −0.86 (95% CI: −0.89; −0.81); R2 = 0.742) (Figure 3b) and a large negative statistically significant correlation for training sessions with >200 TRIMP points (*p* < 0.001; R = −0.67 (95% CI: −0.810; −0.474); R2 = 0.457) (Figure 3c).

### 3.3. Relationships between sRPE and Carbohydrate Consumption During Exercise

**Figure 4:**
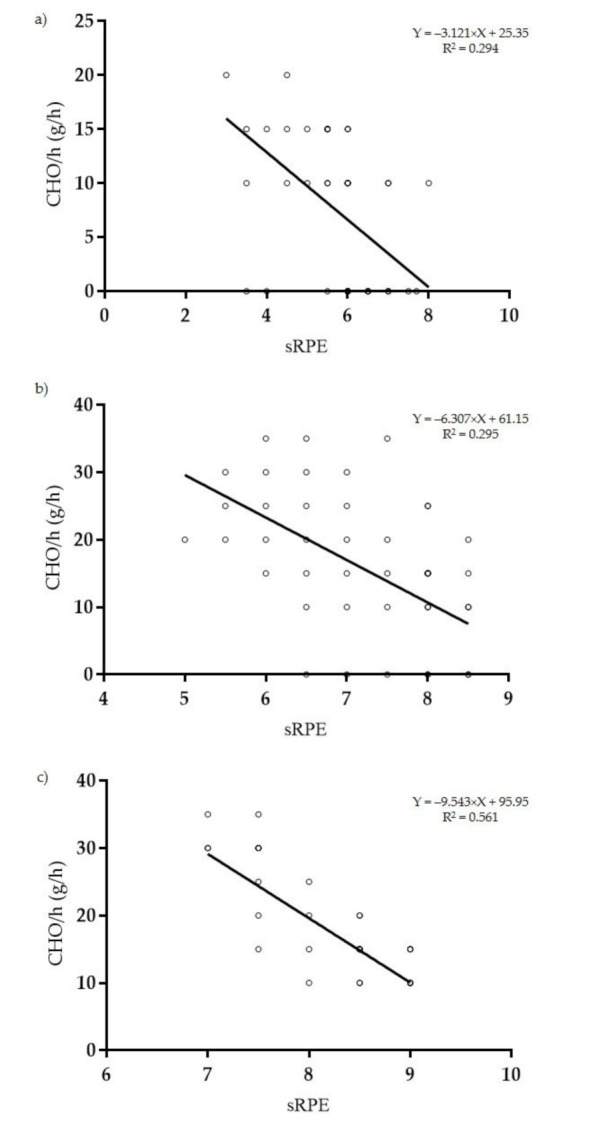
Linear regression between sRPE (AU) and training session carbohydrates consumption (gCHO/h) of training sessions displaying <100 TRIMP points (**a**); 100–200 TRIMP points (**b**) and >200 TRIMP points (**c**).

Correlation analysis with linear regression of sRPE (AU) and training session carbohydrates consumption (gCHO/h), revealed a large negative statistically significant correlation for training sessions with <100 TRIMP points (*p* < 0.001; R = −0.54 (95% CI: −0.70; −0.31); R2 = 0.294) (Figure 4a); a large negative statistically significant correlation for training sessions with 100–200 TRIMP points (*p* < 0.001; R = −0.54 (95% CI: −0.65; −0.41); R2 = 0.295) (Figure 4b) and a very large negative statistically significant correlation for training sessions with >200 TRIMP points (*p* < 0.001; R = −0.75 (95% CI: −0.85; −0.58); R2 = 0.561) (Figure 4c).

### 3.4. Relationships between TRIMP Categories, Fluid Loss and Carbohydrates Consumption During Exercise

Correlation analysis of TRIMP (AU) within the three categories and pre- and post-training ∆ (kg) revealed a small negative non-statistically significant correlation for training sessions with <100 TRIMP points (*p* = 0.26; R = −0.15 (95% CI: −0.40; 0.12); R2 = 0.268), for training session with 100–200 TRIMP points (*p* = 0.16; R = −0.12 (95% CI: −0.27; 0.04); R2 = 0.162), and for training session with >200 TRIMP points (*p* = 0.27; R = −0.17 (95% CI: −0.44; 0.13); R2 = 0.273) (Table 2).

Correlation analysis of TRIMP (AU) within the three categories, and training session carbohydrates consumption (gCHO/h), revealed a small negative non-statistically significant correlation for training sessions with <100 TRIMP points (*p* = 0.08; R = −0.24 (95% CI: −0.47; 0.03); R2 = 0.05), a trivial positive non-significant association for training session with 100–200 TRIMP points (*p* = 0.87; R = 0.01 (95% CI: −0.15; 0.17); R2 = 0.877), and a moderate positive statistically significant association for training session with >200 TRIMP points (*p* = 0.01; R = 0.37 (95% CI: 0.08; 0.60); R2 = 0.136) (Table 2).

## 4. Discussion

This study investigated the relationships between different TRIMP training load categories and sRPE and the role of fluid balance and carbohydrate supply during exercise on perceived fatigue, demonstrating the importance of training load monitoring and nutritional-hydration support for young cyclists’ fatigue management. The investigation involved a team of junior’s category cyclists, monitored as part of their team’s performance and health optimization program that covered an entire season.

The significant differences between training load categories, delineating respectively, recovery, specific adaptations and high load training targets, and the very large correlation that emerged between sRPE and Edward’s TRIMP suggested training and competitions as the main physiological and psychological fatigue perception factors for young athletes as previously described [20,21]. These results support previous observations on the reliability of sRPE as an internal load marker and as a useful non-invasive method to monitor young athletes’ training load [2,4,22]. Thanks to the available non-invasive technologies such as GPS, HR monitoring devices, both Edward’s TRIMP, and sRPE can be easily used as monitoring tools to assess fatigue perception dynamics in relation to training load during the competitive season of young athletes. This can potentially help athletes coaches and team staff to better manage the balance between stress and recovery, that has been linked by previous investigations with injury prevention, athletes’ health, and performance optimization [2,3,4,5,6,9,10,22]. The various benefits of physical activities such as cycling, as opposed to physical inactivity, have been widely demonstrated in different age groups, including adolescents and young athletes [23,24,25,26,27,28,29,30,31,32,33]. However, considering the additional stressors to which these particular age groups are exposed, training load monitoring and management may represent important aspects.

Our results thus suggest the value of training load monitoring and in particular of fatigue perception assessment in young athletes, in order to potentially prevent overload and to ensure performance improvements.

We additionally observed how sRPE was negatively and largely correlated with both, pre- to post-exercise fluid loss (∆) and carbohydrate consumption during exercise (CHO/h), independently from TRIMP training load categories. This confirms once more the importance of hydration status on fatigue perception and suggests the possible role of carbohydrate supply also in low intensity or low volume training (low TRIMP) in young athlete populations [8,11]. During physical activity, carbohydrate availability to the exercising muscle and central nervous system can be compromised due to the fuel cost of the athlete’s training session or competition exceeding the endogenous stores and as a consequence of a lack of external supply [34,35]. The role of carbohydrates in the lower TRIMP training category can be associated with the reduction of fatigue onset and enhanced recovery from training rather than the ergogenic role mainly having an impact at higher training loads levels (i.e., 100–200 and >200 TRIMP point categories) [35,36,37].

This further underlines the role of nutritional education programs for young athletes in order to make them understand the importance of nutritional and fluid support, to preserve health and performance [8].

Kerksick et al. (2018) described water as the most important nutritional ergogenic aid for athletes and that limiting dehydration during exercise is one of the most effective ways to maintain exercise capacity [38]. In addition, it has been reported how dehydration may have a negative impact on mental fatigue accumulation and consequent cognitive performances as well as on psychological status of adolescent and young populations [39]. As suggested by our results and by previous observations, young athletes may experience fluid imbalances if some exercise conditions are met or liquids intake do not satisfy daily requirements, with possible consequences on their physical performance, fatigue accumulation, and health maintenance [11].

**Figure 5:**
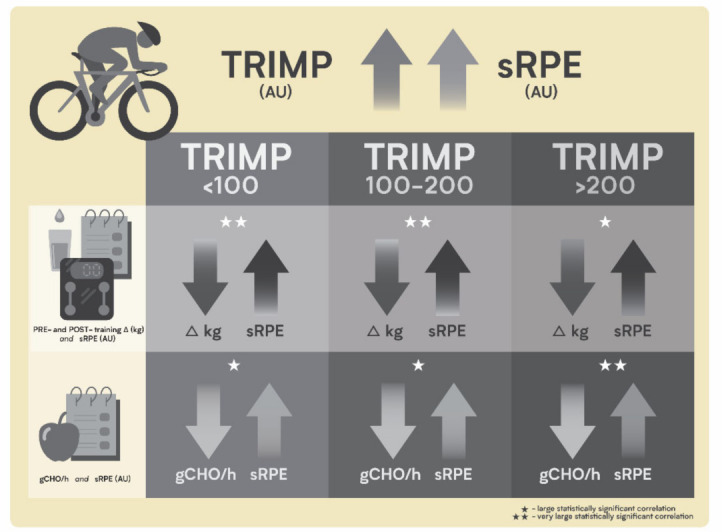
Graphical representation of the inter-relationships between training load markers, fluid balance and carbohydrate supply during exercise. Increased fluid loss (negative ∆) as well as lower or no carbohydrate consumption is related to an increased fatigue perception (sRPE) independently from training load category (TRIMP).

The results of the present study suggest thus the importance of fluid and nutrient supply during low to high training load sessions (e.g., low to high training intensity or volume) in young athletes’ fatigue perception. The small sample size represents one limitation of this study; however, high-level young athletes represent a unique population that is slightly investigated, also due to the difficulty of involvement. This study was therefore carried out thanks to the possibility to work with a complete team (athletes and staff) for the entire duration of a season, which, however, at the same time represented the main limit on the sample size choice. The results of this study show the applicability of a non-invasive and easy-to-apply monitoring strategy. However, although it is recognized as a valid indicator, especially for athletes’ self-assessments by the or for the in-field investigations, the utilization of the pre- and post-training ∆ body mass as a marker of fluid loss is not free from limitations as previously reported (e.g., loss in body mass due to respiratory water losses and substrate oxidation) [40]. The present data described thus a monitoring model suitable for the in-field conditions that cycling team’s staff normally face along the season and underlines the importance of previously described markers in a singular population as the high-level young cyclist (Figure 5).

## 5. Conclusions

Young athletes’ training load monitoring and nutritional support during exercise represent two important aspects for fatigue perception management. The importance of exercise-induced fatigue perception monitoring and the evaluation of a possible contributor of fatigue perception are important tools to support the young athlete. The results emerged from this research are aimed to young athletes’ team staff as an example of a feasible approach to monitor and support the athlete. Future research may aim to further confirm our findings, involving a larger population of young athletes and involving a deeper evaluation on fatigue perception dynamics. 

## Figures and Tables

**Table 1:** Summary of post-hoc analysis results.

Pairwise Comparison	*p*-Value	sRPE (AU) Mean Difference (95% CI)	ES (95% CI)	Interpretation
<100 vs. 100–200	<0.001	−1.34 (−1.61; −1.06)	−1.53 (−1.96; −1.10)	Large
<100 vs. >200	<0.001	−2.58 (−2.97; −2.19)	−2.69 (−3.34; −2.04)	Very Large
100–200 vs. >200	<0.001	−1.24 (−1.48; −1.01)	−1.80 (−2.19; −1.40)	Large

**Table 2:** Results of the correlation analysis between TRIMP within the three different categories, pre- and post-training ∆ (kg) and CHO consumption (g/h).

	TRIMP <100 cat. (AU)			TRIMP 100–200 cat. (AU)			TRIMP > 200 cat. (AU)		
	r	*p*		r	*p*		r	*p*	
∆ (kg)	−0.15	0.26	small	−0.12	0.16	small	−0.17	0.27	small
CHO (g/h)	−0.24	0.08	small	0.01	0.87	trivial	0.37	0.01	moderate

## Data Availability

The data presented in this study are available on request from the corresponding author. The data are not publicly available due to privacy restrictions.
